# Detrimental Effect of Perceived Controlling Behavior from Physical Education Teachers on Students’ Leisure-Time Physical Activity Intentions and Behavior: An Application of the Trans-Contextual Model

**DOI:** 10.3390/ijerph17165939

**Published:** 2020-08-15

**Authors:** Andre Koka, Henri Tilga, Hanna Kalajas-Tilga, Vello Hein, Lennart Raudsepp

**Affiliations:** Institute of Sport Sciences and Physiotherapy, Faculty of Medicine, University of Tartu, Ujula 4 str., 51008 Tartu, Estonia; henri.tilga@ut.ee (H.T.); hanna.kalajas-tilga@ut.ee (H.K.-T.); vello.hein@ut.ee (V.H.); lennart.raudsepp@ut.ee (L.R.)

**Keywords:** basic psychological need frustration, hierarchical model of intrinsic and extrinsic motivation, moderate-to-vigorous physical activity, perceived controlling behavior from teachers, self-determination theory, theory of planned behavior

## Abstract

In the present study, a trans-contextual model was applied to examine the relations between students’ perceptions of controlling behavior from teachers, frustration over their basic psychological needs, autonomous motivation toward physical activity in a physical education context, autonomous motivation toward physical activity in an out-of-school context, beliefs and intentions toward future physical activity, and actual participation in physical activity outside of school. We adopted a three-wave prospective study design in which 234 students aged 11–19 years first completed measures of perceived controlling behavior from teachers, frustration over their basic psychological needs, and autonomous motivation toward physical activity in physical education. One week later, their autonomous motivation, beliefs, and intentions toward physical activity outside of school were measured. Students’ self-reported engagement in physical activity outside of school was assessed another five weeks later. Results of the path analysis revealed a significant and negative indirect effect of perceived controlling behavior from physical education teachers on students’ intention toward physical activity outside of school via the proposed motivational sequence of the trans-contextual model. There was also a significant and negative indirect effect of perceived controlling behavior from physical education teachers on students’ self-reported engagement in physical activity outside of school, mediated by the frustration over their need for competence in physical education. Findings emphasize the importance of decreasing controlling behaviors from teachers in a physical education context so as not to inhibit students’ physical activity behavior in an out-of-school context.

## 1. Introduction

School physical education (PE) has been acknowledged as a beneficial educational context in which lifelong physical activity habits in young people can be promoted [1]. A vast number of studies have demonstrated that adopting the actions and behaviors by PE teachers that are autonomy supportive may facilitate students’ motivation toward school physical activity, but importantly, they may also promote students’ motivation toward physical activity outside of school [2,3,4,5,6,7,8,9,10]. Despite these promising results, according to Sallis et al. [11], about 80% of young people worldwide do not meet the suggested guidelines of the WHO [12]; that is, at least 60 min of moderate-to-vigorous physical activity per day. This has led researchers to identify the factors that may have contributed to this unfortunate situation.

Although teachers adopting behaviors during instructions that are perceived by students as autonomy supportive are most gainful, teachers may fail to do so and adopt actions and behaviors that are perceived by students as controlling instead. Teachers may be perceived as controlling when they overemphasize their own perspective, act intrusively, and pressure students to think, feel, and behave in a particular way [13]. It has been argued that teachers may adopt controlling behaviors during instruction if they feel pressured from above (e.g., school policies, administrators, and parents) or pressured from below (e.g., students’ motivation and behavior in class) [14,15]. Studies in a PE context have shown that students who perceive their teachers as controlling report a greater need frustration [16,17,18,19,20,21] and subsequently lower levels of autonomous motivation and higher levels of controlled motivation and amotivation [22,23,24,25]. In addition, students who perceive their PE teachers to be controlling have also shown lower levels of physical activity outside of school [25]. However, little is known about the psychological processes by which perceived controlling behaviors from teachers in educational contexts relate to students’ motivation and behavior outside of school. The trans-contextual model, developed by Hagger and colleagues [2,3], has been proposed as a feasible framework to explain the relationships between the perceived behaviors from teachers and students’ motivation and behavior toward educational activities across two related contexts; that is, educational and extra-mural contexts. The current study aimed to evaluate an application of the trans-contextual model to explain the processes by which perceived controlling behavior from PE teachers is linked to students’ motivation toward physical activity in PE and outside of school, as well as the beliefs about, intention toward, and actual participation in physical activity outside of school.

**Figure 1:**
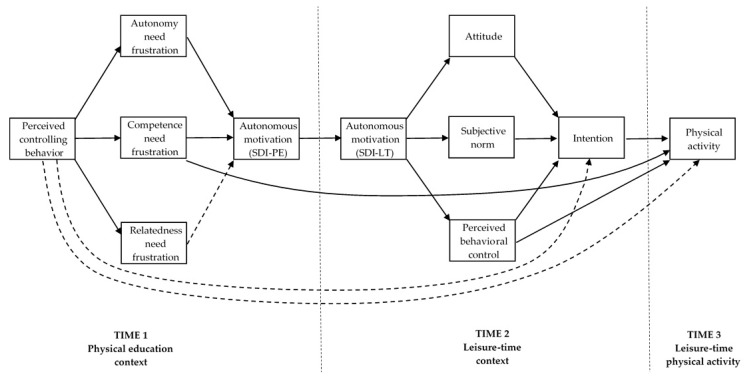
The hypothesized trans-contextual model. The following parameters have been omitted from the figure for clarity reasons: (i) direct paths from past physical activity behavior on all study variables; (ii) error covariances between psychological need frustration variables; and (iii) error covariances between attitude, subjective norm, and perceived behavioral control. The broken lines between constructs indicate direct effects that are assumed to be non-significant. SDI-PE = self-determination index in a physical education context; SDI-LT = self-determination index in a leisure-time context.

The trans-contextual model derives its hypotheses from self-determination theory [26,27], the hierarchical model of intrinsic and extrinsic motivation [28], and the theory of planned behavior [29,30]. The key hypotheses of the model have been supported in many studies conducted in PE and leisure-time physical activity contexts [2,3,5,6,9], as well as in a recent meta-analysis [10]. Particularly, these studies have supported the effects of perceived autonomy support on autonomous motivation in PE, the trans-contextual effect of autonomous motivation in PE on autonomous motivation to take part in physical activity outside of school, and links between autonomous motivation and beliefs about, intentions toward, and actual physical activity participation outside of school. In brief, tests of the trans-contextual model so far have provided extensive empirical evidence on the role of students’ perceptions of positive teaching behavior (i.e., an autonomy-supportive interpersonal behavior) exhibited by PE teachers on students’ educational outcomes both in and out of the classroom. In the present study, we explicitly address the role of students’ perceptions of negative teaching behavior (i.e., a controlling interpersonal behavior) exhibited by PE teachers on students’ educational outcomes both in and out of the classroom. Therefore, in the next sections, we outline the key hypotheses of the trans-contextual model in terms of controlling behavior from PE teachers perceived by students and its relations with students’ motivation, beliefs about, intentions toward, and actual physical activity participation outside of school. A diagram of the trans-contextual model predictions is presented in Figure 1.

Research in PE guided by self-determination theory [26,27] has demonstrated that students felt frustration over their basic psychological needs for autonomy, competence, and relatedness [16,17,18,19,20,21], and subsequently lower levels of autonomous forms of motivation and higher levels of controlled forms of motivation and amotivation in classes [22,23,24,25], if they perceived their teachers to exhibit controlling behaviors such as intimidation, controlling use of praise, and negative conditional regard. The proposed effect of perceived controlling behavior from PE teachers on students’ motivation, mediated by experiences of frustration over their basic psychological needs in the classroom, forms the first three hypotheses of the trans-contextual model in the current study. Specifically, it was expected that perceived controlling behavior from PE teachers would have significant direct and positive effects on the frustration over their basic psychological needs for autonomy, competence, and relatedness (H_1_). In line with the study by Koka et al. [25], who found that frustration of the need for autonomy and competence, but not relatedness, had direct effects on various forms of motivational regulation in PE, it was expected that frustration over the need for autonomy and competence would have significant direct and negative effects on autonomous motivation toward PE (H_2_). The hypothesized significant and negative relation of perceived controlling behavior from PE teachers with autonomous motivation toward PE would be mediated by their frustration over their psychological needs for autonomy and competence (H_3_).

A review and meta-analysis of the research using the trans-contextual model as the theoretical framework have demonstrated that students’ autonomous motivation toward physical activity in PE is related to their autonomous motivation toward physical activity outside of school [8,10]. This motivational transfer across contexts is derived from Vallerand’s [28] hierarchical model of intrinsic and extrinsic motivation, claiming that individuals who experience autonomous motivation while engaging in educational activities at school may likely develop autonomous motivation toward educational activities in other related contexts, such as the leisure-time context. This proposition forms the fourth hypothesis of the trans-contextual model in the current study; that is, students’ autonomous motivation toward PE would be significantly and positively related to students’ autonomous motivation toward physical activity outside of school in their leisure time (H_4_).

An important addition to the initial formulation of the trans-contextual model by studies of Barkoukis et al. [6] and Gonzales-Cutre et al. [9] was the autonomous motivation toward PE as a mediator of the relationship between perceived satisfaction of basic psychological needs in PE and autonomous motivation toward leisure-time physical activity. It was found that the perceived satisfaction of the need for autonomy and competence, but not relatedness, in PE, had significant indirect effects on the autonomous motivation toward leisure-time physical activity, mediated by autonomous motivation toward PE. Koka et al. [25] found that different forms of motivational regulation toward PE mediated the effects of perceived frustration over the need for autonomy and competence in PE, but not relatedness, on the respective forms of motivational regulation toward leisure-time physical activity. Based on this, we formed our fifth hypothesis, which states that autonomous motivation toward PE functions as a mediator of the relationship between perceived frustration over the need for autonomy and competence in PE and autonomous motivation toward leisure-time physical activity (H_5_).

According to the theory of planned behavior [29,30], individuals’ intention to engage in a particular activity in the future is dependent on one’s beliefs about that activity, including a subjective evaluation of the activity (i.e., attitude), perceptions of appropriateness and prevalence of the activity by significant others (i.e., subjective norms), and perceptions of controllability over the activity (i.e., perceived behavioral control). It has been supported that students who are autonomously motivated toward educational activities more likely form positive attitudes and perceived control toward these activities, and, in turn, a higher intention to perform these activities in the future [31]. The trans-contextual model, therefore, proposes that the relationship between motivation and intention to engage in a particular activity is mediated by the immediate belief-based determinants of intention; that is, attitude, subjective norms, and perceived behavioral control [8,10]. On this basis we formed the next set of hypotheses, i.e., Hypotheses 6–8 of the current study. First, it was expected that autonomous motivation toward physical activity at leisure time would have direct and positive effects on attitude and perceived behavioral control, whereas no effect or negative effect on subjective norms (H_6_) as the latter indicate students’ beliefs of what significant others like teachers want them to do and are therefore interpreted as controlling. Secondly, it was hypothesized that attitude, subjective norms, and perceived behavioral control would have direct and positive effects on intention to participate in leisure-time physical activity (H_7_). Thirdly, it was expected that autonomous motivation toward leisure-time physical activity is related positively to intention through the mediation of attitude and perceived behavioral control, whereas the indirect effect through subjective norms would be non-significant or negative (H_8_). The final proposition of the theory of planned behavior is that behavioral intention is the proximal predictor of actual behavior [29,30]. In addition to the direct effect of intention on actual behavior, the theory of planned behavior proposes that if the individual perceptions of control over the behavior are realistic, then the perceived behavioral control is also expected to predict the behavior directly. As these proposed effects have found extensive empirical support [10], it was hypothesized that both the intention to be physically active in leisure time and the perceived behavioral control over the physical activity behavior will have direct positive effects on students’ physical activity in their leisure time (H_9_). 

Based on the results of the recent study by Koka et al. [25], an additional direct effect from perceived frustration over the need for competence in PE on students’ physical activity at leisure time should be considered. This is because students’ experiences of frustration over their need for competence in PE was found to have a direct negative effect on students’ leisure-time physical activity. Therefore, a direct and negative effect of perceived frustration over the need for competence in PE on students’ physical activity participation in leisure time formed the tenth hypothesis of the present study (H_10_).

The final set of hypotheses of the current study is related to testing the effects of an independent variable (i.e., perceived controlling behavior from PE teachers) on the key dependent variables of the model (i.e., students’ intentions to participate in leisure-time physical activity and actual physical activity behavior) via the mediation of the entire proposed motivational sequence of the model. As presented in a recent meta-analysis by Hagger and Chatzisarantis [10], previous tests of the trans-contextual model revealed significant and positive indirect effects of perceived autonomy support from PE teachers on students’ intentions toward leisure-time physical activity (β = 0.10, *p* < 0.001) and actual participation in physical activity (β = 0.05, *p* = 0.035) via the mediation of the proposed motivational sequence of the model. Whereas the focus of the present study was on negative teaching behavior, it was hypothesized that there would be a significant and negative indirect effect of the perceived controlling behavior from PE teachers on students’ intentions to participate in leisure-time physical activity, mediated by the proposed motivational sequence of the model (H_11_). The relationship between the perceived controlling behavior from PE teachers and students’ actual physical activity behavior was expected to be negative and mediated via two pathways: (i) via the entire proposed motivational sequence of the model; and (ii) via the perceived frustration of the need for competence in PE alone (H_12_).

To recapitulate, a vast number of studies have supported the key propositions of the trans-contextual model, including the effects of students’ perceptions of autonomy support from PE teachers on autonomous motivation toward PE, the transfer of autonomous motivation across PE and leisure-time physical activity contexts, and links between autonomous motivation and beliefs about, intentions toward, and actual participation in leisure-time physical activity [2,3,4,5,6,8,9,10]. The previous research that adopted the trans-contextual model have thus exclusively focused on the role of perceived positive behavior from PE teachers on students’ learning outcomes, both in a PE and leisure-time physical activity context. There are only a few studies that have demonstrated the unfavorable role of perceived negative behavior from PE teachers on students’ leisure-time physical activity [25,32,33,34]. To the best of our knowledge, however, no studies to date have applied the entire motivational sequence proposed in the trans-contextual model in investigating the relationship between perceived negative (i.e., controlling) behavior exhibited by teachers in an educational context (i.e., PE context) and students’ actual participation in physical activity outside of school. The present study, therefore, aimed to contribute to the existing knowledge by examining whether students’ perceptions of controlling behavior from PE teachers have detrimental effects on their motivation toward physical activity in PE, but most importantly, students’ beliefs about, motivation and intention toward, and actual physical activity participation in a leisure-time context. 

## 2. Materials and Methods 

### 2.1. Participants and Procedure

Participants were 285 students aged 11–19 years studying in eight state schools. Fifty-one students out of 285 dropped out from the study due to their absences on the testing day at Time 2 or Time 3, which resulted in a final sample size of 234 participants (104 boys and 130 girls; *M_age_* = 15.29 years, *SD* = 2.06). Specifically, 100 were secondary school students (i.e., 7th, 8th, and 9th graders; 41 boys and 59 girls; *M_age_* = 13.18 years, *SD* = 0.97) and 134 were high school students (i.e., 10th, 11th, and 12th graders; 63 boys and 71 girls; *M_age_* = 16.86 years, *SD* = 0.94). Students were eligible for inclusion if they were 7th–12th-grade students without restrictions on their participation in physical activity. We first obtained the consent for data collection from the schools’ administration. Then the informed consent from students and their parents was received via a consent form sent home. The parental permission was considered approved if they did not send the form back to the school. None of the forms were sent back. At the first wave of data collection the participants were briefed that they would be asked to complete another two series of questionnaires over the next coming weeks.

In line with the initial validation of the trans-contextual model [2], the present study adopted a prospective design in which psychological variables were assessed at three points in time. In the first wave of data collection (Time1), the self-report measures of the perceived teachers’ controlling behavior, perceived frustration over the needs for competence, autonomy, and relatedness, as well as different forms of motivational regulations in a PE context were administered. In the second wave of data collection (Time 2), which took place one week later, different forms of motivational regulations toward leisure-time physical activity, components of the theory of planned behavior, as well as the incidence of leisure-time physical activity in the past 6 months were measured. In line with previous studies [2,3,5,7,25], a latency period of one week between the Time 1 and Time 2 points of data collection was employed to reduce the common method variance associated with the use of similar methods to assess different forms of motivational regulations in different contexts (i.e., PE and leisure-time physical activity contexts). In the third wave of data collection (Time 3), which took place another five weeks later, participants’ self-reported physical activity behavior was measured. According to Hagger et al. [35], a five-week period between the Time 2 and Time 3 data collection would permit a longer-range prediction of physical activity behavior. Participants’ gender and date of birth were used as matching principles for prospective responses to the questionnaires. The students completed questionnaires at all three points in time in the regular classroom. The study was anonymous to preserve confidentiality. The approval of the design and procedures of the present study was obtained from the Research Ethics Committee of the University of Tartu, Estonia (273/T-5). It should be noted that part of the data were previously published to investigate the mediating role of perceived frustration over basic psychological needs and different forms of motivation in the relationship between perceived controlling behavior from PE teachers and objectively measured physical activity of adolescents in Koka et al. [25]. The latter study, however, did not investigate the contributing role of constructs from the theory of planned behavior, such as attitude, perceived behavioral control, subjective norms, and intention towards physical activity in explaining the relations between motivational processes in PE and leisure-time physical activity behavior.

### 2.2. Measures

Unless mentioned otherwise, responses to the questionnaire items were provided on a 7-point Likert scale ranging from 1 (strongly disagree) to 7 (strongly agree).

#### 2.2.1. Perceived Controlling Behavior from PE Teachers 

Students’ perceptions of controlling behaviors from PE teachers were assessed using three three-item subscales (intimidation, negative conditional regard, and controlling use of praise) from the Controlling Coach Behaviors Scale (CCBS) [36]. The CCBS has been modified to a PE context [16]. Items were preceded by the common stem (“My PE teacher …”), followed by items such as “… is less supportive of me when I do not exercise and perform well” (negative conditional regard), “… only uses praise to make me exercise harder” (controlling use of praise), and “… uses the threat of punishment to keep me in line during lesson” (intimidation). We excluded the subscale of excessive controlling behavior (e.g., “My PE teacher tries to control what I do during my free time out of school”) from the current study as previous studies [16] have shown it to be irrelevant to the PE context. Previous research has shown evidence to support the factorial validity and reliability of the PE-modified version of the CCBS as well as its tenability to adolescent samples [19,24,32,37].

#### 2.2.2. Perceived Frustration of Basic Psychological Needs 

Students’ perceptions of their frustration over the need for autonomy, competence, and relatedness were assessed using the subscales from the Basic Psychological Need Satisfaction and Frustration Scale [38], modified to a PE context by Haerens et al. [22]. Items were preceded by the common stem (“During the PE lesson…”), followed by items such as “…I felt forced to do many exercises I wouldn’t choose to do” (frustration over the need for autonomy), “…I felt disappointed with many of my performances” (frustration over the need for competence), and “…I felt that class members who are important to me were cold and distant towards me” (frustration over the need for relatedness). Each subscale consists of four items. Studies in PE with students of similar age to the current study have provided evidence for the high internal consistency and factorial validity of the scale [19,22].

#### 2.2.3. Motivational Regulations in Physical Education and Leisure-Time Contexts 

Students’ different types of motivational regulations in a PE context were measured using the Perceived Locus of Causality (PLOC) scale [39]. Items were preceded by the common stem (“I take part in PE...”), followed by different reasons such as intrinsic motivation (e.g., “...because PE is fun”), identified regulation (e.g., “...because it is important to me to do well in PE”), introjected regulation (e.g., “...because I would feel bad if the other students thought that I am not good at PE”), external regulation (e.g., “...because that’s the rule”), and amotivation (e.g., “…but I really do not know why”). All subscales consist of four items. The reliability and validity of the PLOC scale has found extensive support in studies with adolescents [7,40,41]. 

Students’ different types of motivational regulations in a leisure-time context were assessed using the Behavioral Regulations in Exercise Questionnaire-2 (BREQ-2) [42]. Items were preceded by the common stem (“I take part in active sports and/or moderate-to-vigorous physical activities in my spare time...”), followed by different reasons such as intrinsic motivation (e.g., “...because it is fun”), identified regulation (e.g., “...because I value the benefits of physical activities”), introjected regulation (e.g., “…because I will feel guilty if I do not”), external regulation (e.g., “...because other people say I should”), and amotivation (e.g., “…but I really do not know why”). All subscales consist of four items. The BREQ-2 has been used among students of a similar age to the current study [7].

#### 2.2.4. Theory of Planned Behavior Constructs 

The items of the theory of planned behavior constructs were created according to the reported guidelines [43]. Students’ behavioral intentions were assessed by two items (e.g., “I intend to do active sports and/or vigorous physical activities during my leisure time in the next 5 weeks”). Attitudes were assessed in response to the common stem: “For me participating in active sports and/or vigorous physical activities during my leisure time in the next 5 weeks is…”. Responses were assessed on three 7-point semantic differential scales with the following bipolar adjectives: unenjoyable–enjoyable, bad–good, and useful–useless. Subjective norms were assessed by two items (e.g., “Most people important to me think that I should do active sports and/or vigorous physical activities during my leisure time in the next 5 weeks”). Perceived behavioral control was assessed by two items (e.g., “I feel in complete control over whether I do active sports and/or vigorous physical activities in my leisure time in the next 5 weeks”) on 7-point Likert-type scales ranging from 1 (no control) to 7 (complete control). Previous studies have shown measures of the theory constructs in school children to be valid and reliable [2,3,4,5].

#### 2.2.5. Self-Reported Leisure-Time Moderate-to-Vigorous Physical Activity Behavior

Students’ physical activity participation was measured using the short form of the International Physical Activity Questionnaire (IPAQ) [44], which was modified to make explicit reference to leisure-time physical activity. The short form of IPAQ collects information on frequency (days) and time (minutes) spent on vigorous physical activity, moderate physical activity, walking, and sitting over the past 7 days. In the present study only items recording the frequency and time spent on vigorous and moderate physical activity was used. Weekly minutes of vigorous and moderate physical activity were calculated separately by multiplying the number of days per week by the duration on an average day. The total minutes/week for each activity intensity was then multiplied with the designated metabolic equivalent of task (MET) value, resulting in a physical activity estimate expressed in MET-minutes/week. Estimates for moderate and vigorous physical activities were summed to create a score for students’ moderate-to-vigorous physical activity. The IPAQ has demonstrated fairly good measurement properties for monitoring physical activity among adolescents [45].

#### 2.2.6. Past Moderate-to-Vigorous Physical Activity Behavior

Students’ past moderate-to-vigorous physical activity was assessed by one item that asked the participants to report how often they had been doing active sports and/or moderate-to-vigorous physical activities during the last 6 months. Participants responded on a 6-point scale anchored by 1 (not at all) to 6 (most of the days per week). This measure has been previously used in many studies to estimate the frequency of past physical activity behavior among adolescents [2,3,5,25]. 

### 2.3. Data Analysis

The data were analyzed using SPSS Version 23 (IBM corp., Armonk, NY, USA) and AMOS Version 23 (IBM corp., Armonk, NY, USA) statistical software. Prior to the analyses, the data were checked for data entry errors and missing values. In the preliminary analyses, the descriptive statistics, Cronbach’s alphas, and zero-order correlations between the study variables were calculated. Then, depending on the distribution of the data, the Mann–Whitney U-test or Independent Samples T-test was used to evaluate whether there were mean differences among the study variables measured at Time 1 between participants who completed the study measures at all three points of data collection and those who did not. In addition, the mean differences were measured on all study variables between the 7th–9th graders and 10th–12th graders. 

In the main analyses, the hypothesized relationships among the study variables were examined using path analysis with the maximum likelihood estimation method. Prior to the path analysis, composite scores for each study variable, except for perceived controlling behavior, autonomous motivation in a PE and leisure-time context, and self-reported physical activity behavior, were computed by averaging the items of each scale. A single construct of perceived controlling behavior from PE teachers was calculated as a composite of the negative conditional regard, controlling use of praise, and intimidation subscales weighted by the second-order factor loadings from the confirmatory factor analysis. The latter procedure was used to enable each subscale to make a unique contribution to the perceived controlling behavior construct [46]. In line with previous studies, different motivational regulations both in the PE and leisure-time contexts were combined into a single index by computing a self-determination index (SDI) reflecting autonomous motivation [3,47]. Consequently, the averaged score of each motivational regulation was weighted as follows: intrinsic motivation (+3), identified regulation (+2), introjected regulation (–1), extrinsic regulation (–2), and amotivation (−3), and a single SDI in the PE and leisure-time contexts were computed based on the weighted composite of these scores. The construct of the self-reported physical activity behavior was treated as described in the measures section. Past physical activity was included as a control variable that predicted all other variables in the hypothesized model.

The adequacy of the fit of the hypothesized model was assessed using the following indices: Comparative Fit Index (CFI), Non-Normed Fit Index (NNFI), and Root Mean Squared Error of Approximation (RMSEA) with its 90% confidence intervals (CI_90_). The values for CFI and NNFI ≥ 0.95 were considered indicative of an acceptable fit [48]. The values ≤ 0.06 for RMSEA, with values ≤ 0.10 for its upper limit of CI_90_, indicate a good fit [49]. In line with the recommendations by Preacher and Hayes [50], the path analysis was accompanied with bootstrapping analysis to determine the standardized parameter estimates and the significance of the indirect effects in the model. If the 95% confidence interval (CI_95_) for the indirect effect does not contain the null value, then it can be concluded that the indirect effect is statistically significant.

## 3. Results

### 3.1. Preliminary Analyses

Data screening showed that all the study variables had less than 5% missing values. The non-significant Little’s [51] test of missing completely at random revealed that the data were missing completely at random (χ^2^ = 3999.37, df = 3913, *p* > 0.05). Thus, the Expectation Maximization algorithm was used to impute the missing values.

The Mann–Whitney U-test or Independent Samples T-test indicated that participants who dropped out from the study after the completion of measures at Time 1 scored significantly higher on perceived negative conditional regard (*M* = 3.22, *SD* = 1.40 vs *M* = 2.64, *SD* = 1.32, *t* = 2.77, *p* < 0.01) and intimidation behavior (*M* = 2.19, *SD* = 1.30 vs *M* = 1.80, *SD* = 1.02, *U* = 4945.50, *z* = −1.97, *p* < 0.05) compared with participants who were retained for the analyses. In addition, participants who dropped out from the study were significantly younger (*M*_age_ = 13.47, *SD* = 1.38 vs *M*_age_ = 15.29, *SD* = 2.06, *t* = 7.73, *p* < 0.001) than participants who were retained for the analyses. There was no significant difference in the distribution of gender (χ^2^ = 1.84, df = 1, *p* > 0.05) between the participants who dropped out from the study and those who did not. Furthermore, there were no significant differences in the study variables (*t*s = 0.17–1.90, *p*s > 0.06), except for behavioral intention (*t* = 2.67, *p* < 0.01) between the 7th–9th graders and 10th–12th graders. Specifically, the 10th–12th graders (*M* = 5.72, *SD* = 1.40) scored significantly higher on behavioral intention compared with the 7th–9th graders (*M* = 5.22, *SD* = 1.46). There was no significant difference in the distribution of gender (χ^2^ = 0.84, df = 1, *p* > 0.05) between the 7th–9th graders and 10th–12th graders.

Table 1 presents the descriptive statistics and Cronbach’s alpha coefficients for all the measures. The intercorrelations are presented in Table 2.

### 3.2. Main Analyses

**Figure 2:**
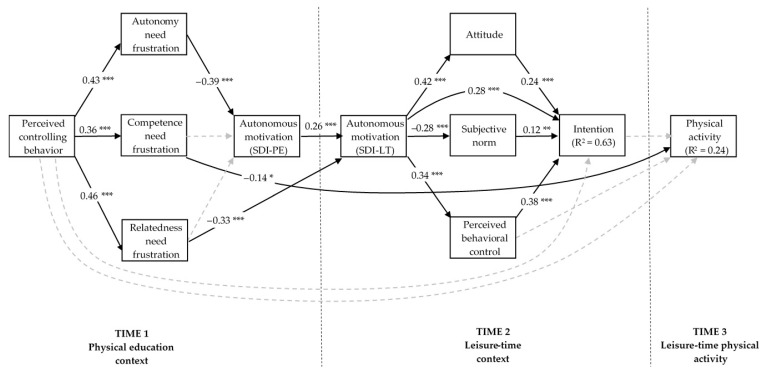
Standardized path estimates for the hypothesized relations among the trans-contextual models. The faint broken lines are indicative of nonsignificant paths. The following parameters have been omitted from the figure for clarity reasons: (i) direct paths from past physical activity behavior on all study variables; (ii) error covariances between the psychological need frustration; and (iii) error covariances between attitude, subjective norms, and perceived behavioral control. Past physical activity had significant direct effects on autonomy need frustration (−0.12 *), competence need frustration (−0.19 ***), SDI-PE (0.19 ***), SDI-LT (0.34 ***), attitude (0.17 **), subjective norms (0.23 ***), perceived behavioral control (0.30 ***), and physical activity behavior (0.42 ***). SDI-PE = self-determination index in a physical education context; SDI-LT = self-determination index in a leisure-time context. * *p* < 0.05; ** *p* < 0.01; *** *p* < 0.001.

Results of the path analysis revealed that the hypothesized model exhibited a poor data fit (χ^2^ = 114.36, df = 31; CFI = 0.92; NNFI = 0.83; RMSEA = 0.107, CI_90_ for RMSEA range = 0.087–0.129). The inspection of the modification indices suggested to add two direct paths from (i) perceived frustration over the need for relatedness to autonomous motivation in a leisure-time context; and (ii) autonomous motivation in a leisure-time context to intention. The model was reanalyzed that yielded an acceptable fit to the data (χ^2^ = 53.63, df = 29; CFI = 0.98; NNFI = 0.95; RMSEA = 0.060, CI_90_ for RMSEA range = 0.034–0.085). The standardized path coefficients are presented in Figure 2. Table 3 presents the standardized indirect effects along with their CI_95_.

**Hypothesis** **1.** As predicted, results revealed that perceived controlling behavior from PE teachers had direct and positive effects on frustration over the need for autonomy (β = 0.43; CI_95_ = 0.31, 0.53; *p* < 0.001), competence (β = 0.36; CI_95_ = 0.24, 0.47; *p* < 0.001), and relatedness (β = 0.46; CI_95_ = 0.31, 0.58; *p* < 0.001).

**Hypothesis** **2.** Perceived frustration of the need for autonomy (β = −0.39; CI_95_ = −0.54, −0.24; *p* < 0.001), but not competence (β = −0.14; CI_95_ = −0.29, 0.02; *p* > 0.05), had a direct and negative effect on autonomous motivation in PE, thus supporting the hypothesis only partially.

**Hypothesis** **3.** Perceived controlling behavior from teachers was expected to have a significant and negative indirect effect on autonomous motivation in PE mediated by the perceived frustration over the need for autonomy and competence. The total indirect effect of perceived controlling behavior from teachers (β = −0.24; CI_95_ = −0.34, −0.15; *p* < 0.001) on autonomous motivation in PE was significant. Tests of specific indirect effects revealed that the indirect effect of perceived controlling behavior on autonomous motivation in PE via perceived autonomy need frustration (B = −2.25; CI_95_ = −3.67, −1.20; β = −0.17; *p* < 0.001) was significant, but not via competence need frustration (B = −0.67; CI_95_ = −1.54, 0.07; β = −0.05; *p* > 0.05). This indicated that perceived autonomy need frustration alone functioned as a mediator of the relationship between perceived controlling behavior and autonomous motivation in PE, thus supporting Hypothesis 3 only partially.

**Hypothesis** **4.** Autonomous motivation in PE had a direct and positive effect on autonomous motivation in a context of leisure-time physical activity (β = 0.26; CI_95_ = 0.15, 0.37; *p* < 0.001), as expected.

**Hypothesis** **5.** Autonomous motivation in PE mediated the relationship between autonomy need frustration (β = −0.10; CI_95_ = −0.16, 0.06; *p* < 0.001), but not competence need frustration (β = −0.04; CI_95_ = −0.09, 0.00; *p* > 0.05), and autonomous motivation in a context of leisure-time physical activity. Hypothesis 5, therefore, was supported partially. As was suggested by the modification indices, relatedness need frustration (β = −0.33; CI_95_ = −0.43, −0.22; *p* < 0.001) demonstrated a significant direct and negative effect on autonomous motivation in a context of leisure-time physical activity.

**Hypothesis** **6.** The results revealed that, as expected, autonomous motivation in a context of leisure-time physical activity had direct and positive effects on attitude (β = 0.42; CI_95_ = 0.29, 0.55; *p* < 0.001) and perceived behavioral control (β = 0.34; CI_95_ = 0.20, 0.48; *p* < 0.001), whereas direct and negative effect on subjective norms (β = −0.28; CI_95_ = −0.42, −0.13; *p* < 0.001).

**Hypothesis** **7.** Attitude (β = 0.24; CI_95_ = 0.11, 0.38; *p* < 0.001), subjective norms (β = 0.12; CI_95_ = 0.03, 0.22; *p* < 0.01), and perceived behavioral control (β = 0.38; CI_95_ = 0.24, 0.53; *p* < 0.001) had direct and positive effects on intention, as hypothesized.

**Hypothesis** **8.** It was expected that autonomous motivation in a leisure-time physical activity context will have a significant indirect effect on intention through the mediation of attitude, subjective norms, and perceived behavioral control. The total indirect effect of autonomous motivation in a leisure-time physical activity context (β = 0.20; CI_95_ = 0.10, 0.31; *p* < 0.001) on intention was significant. Tests of specific indirect effects revealed that indirect effects of autonomous motivation in a leisure-time physical activity context on intention via attitude (B = 0.02; CI_95_ = 0.01, 0.03; β = 0.10; *p* < 0.001) and perceived behavioral control (B = 0.02; CI_95_ = 0.01, 0.04; β = 0.13; *p* < 0.001) were significant and positive, whereas significant and negative via subjective norms (B = −0.01; CI_95_ = −0.01, −0.00; β = −0.03; *p* < 0.01). However, there was also a significant and positive direct effect of autonomous motivation in a leisure-time physical activity context on intention (β = 0.28; CI_95_ = 0.13, 0.43; *p* < 0.001), revealing that any mediation of the effect of autonomous motivation in a leisure-time physical activity context on intention would be partial, thus supporting Hypothesis 8 only partially. The total effect of autonomous motivation in a leisure-time physical activity context on intention (β = 0.47; CI_95_ = 0.35, 0.59; *p* < 0.001), therefore, was both direct and indirect via attitude, subjective norms, and perceived behavioral control.

**Hypothesis** **9.** The results revealed no significant direct effect from intention (β = 0.08; CI_95_ = −0.06, 0.22; *p* > 0.05) nor perceived behavioral control (β = −0.04; CI_95_ = −0.17, 0.09; *p* > 0.05) to students’ self-reported leisure-time moderate-to-vigorous physical activity. Given that the effect of intention on self-reported leisure-time moderate-to-vigorous physical activity was not statistically significant, intention did not mediate the effects of attitude (indirect effect β = 0.02; CI_95_ = −0.01, 0.06; *p* > 0.05), subjective norms (indirect effect β = 0.01; CI_95_ = −0.00, 0.04; *p* > 0.05), and perceived behavioral control (indirect effect β = 0.03; CI_95_ = −0.02, 0.09; *p* > 0.05) on students’ self-reported leisure-time moderate-to-vigorous physical activity, leading us to a rejection of these hypothesized mediation effects.

**Hypothesis** **10.** As hypothesized, perceived frustration over the need for competence in PE (β = −0.14; CI_95_ = −0.25, −0.03; *p* < 0.05) had a direct and negative effect on students’ self-reported leisure-time moderate-to-vigorous physical activity.

**Hypothesis** **11.** Consistent with the hypothesis, the results indicated a statistically significant and negative total indirect effect of perceived controlling behavior from PE teachers (β = −0.10; CI_95_ = −0.17, −0.06; *p* < 0.001) on students’ intention to be physically active in leisure time mediated by the proposed motivational sequence. The significance of the specific indirect effects from perceived controlling behavior from PE teachers to intention was estimated next based on the significant single paths shown in Figure 2. As presented in Table 4, six out of eight statistically significant specific indirect effects of perceived controlling behavior from PE teachers on intention were negative, including the indirect effects of perceived controlling behavior from PE teachers on intention (a) via the autonomy need frustration and autonomous motivation in both contexts; (b) via the autonomy need frustration, autonomous motivation in both contexts, and attitude and perceived behavioral control, respectively; (c) via the relatedness need frustration and autonomous motivation in a leisure-time context; and (d) via the relatedness need frustration, autonomous motivation in a leisure-time context, and attitude and perceived behavioral control, respectively. Two of the eight statistically significant specific indirect effects of perceived controlling behavior from PE teachers on intention, on the other hand, were positive, including the indirect effects of perceived controlling behavior from PE teachers on intention (a) via the autonomy need frustration, autonomous motivation in both contexts, and subjective norms; and (b) via the relatedness need frustration, autonomous motivation in a leisure-time context, and subjective norms.

**Hypothesis** **12.** The results revealed a statistically significant and negative total indirect effect of perceived controlling behavior from PE teachers on students’ self-reported leisure-time moderate-to-vigorous physical activity (β = −0.05; CI_95_ = −0.11, −0.01; *p* < 0.01). Given that perceived frustration over the need for competence in PE was the sole direct predictor of students’ self-reported physical activity, a test of the specific indirect effect indicated that the majority of the indirect effects of the perceived controlling behavior from PE teachers on physical activity was mediated by perceived frustration over the need for competence in PE (B = −143.86; CI_95_ = −286.91, −33.75; β = −0.05; *p* < 0.01) and not by the proposed motivational sequence. Therefore, the hypothesis was supported only partially.

## 4. Discussion

The present study examined the effects of students’ perceptions of controlling behavior from PE teachers on students’ autonomous motivation toward activities in PE, but most importantly, on autonomous motivation toward physical activity, beliefs about, intention toward, and actual participation in physical activity in an out-of-school context. In doing so, we adopted the trans-contextual model, an integrated multi-theory approach proposed by Hagger and colleagues [2,3,8,10]. Findings supported most of the hypotheses proposed by the self-determination theory [26,27], hierarchical model of intrinsic and extrinsic motivation [28], and the theory of planned behavior [29,30], the constituent theories of the trans-contextual model of motivation. Results of the present study showed that the controlling behavior from the teachers as perceived by the students in PE classes had a detrimental effect on the students’ intention toward physical activity behavior outside of school, because it led to higher levels of student perceptions of frustration over the need for autonomy in PE and, in turn, lower levels of autonomous motivation for both in- and out-of-school physical activity, as well as belief-based constructs from the theory of planned behavior, such as attitude and perceived behavioral control. Furthermore, the results revealed that students’ perceptions of controlling behavior from their teachers in PE had a detrimental effect on the students’ actual participation in physical activity outside of school as it elicited students’ feelings of frustration over their need for competence while engaging in PE classes at school.

While many of the proposed effects found support, there were some effects that did not support predictions. The first ones that deserve attention are the null effect of perceived frustration over the need for competence in PE on autonomous motivation toward PE, and the perceived frustration over the need for competence not functioning as an expected mediator between perceived controlling behavior from teachers and students’ autonomous motivation toward PE. The contributing effect of competence need frustration in forming the most controlled forms of motivation, such as external regulation in PE, has been found previously [25]. The use of a self-determination index in the present study may have masked the unique effect of the perceived frustration of the need for competence on students’ motivation toward PE. Future research examining the role of perceived controlling behavior from PE teachers on students’ educational outcomes within the framework of a trans-contextual model would do well to make the distinction between different forms of motivational regulation to determine specific cross-contextual pathways in line with the initial test of the trans-contextual model [2]. The analysis of such a complex model, however, requires a larger sample size so that the required number of cases per parameter estimates would have been met. Our sample size was insufficient to estimate such a model.

The most prominent among the null effects found in the present study were the non-significant effects of intention on physical activity behavior, and of perceived behavioral control on physical activity behavior. The failure to find significant direct effects of intention, and of perceived behavioral control on physical activity behavior that have been shown in many previous tests of trans-contextual model [8,10] may be likely due to issues of measurements used in the current study. Specifically, the items used to assess students’ intention toward physical activity, and perceptions of control over the leisure-time physical activity, referred to doing physical activities in the next 5 weeks. The short form of the IPAQ, however, collected information on frequency and time spent on physical activity over the past 7 days. So, physical activity behavior was assessed only over a short period of time (i.e., one week) while the measures of intention as well as perceived behavioral control referred to the longer time period (i.e., next five weeks). It has been suggested that items of construct such as intention should closely correspond with behavioral measures in terms of time [29]. Such a necessary correspondence in terms of time period between the measure of physical activity behavior and measures of intention and perceived behavioral control over which the students’ self-reports were taken was not insured, and that should be considered as one of the major limitations of the present study.

Another possible reason for a nonsignificant intention–behavior relationship found in the current study might be the past physical activity behavior that was entered into the model as a control variable predicting all other variables, including the future physical activity behavior. It has been proposed that the more individuals have engaged in a particular behavior in the past, the less important the intention is in predicting that behavior in the future [52]. As there was a relatively strong past–future behavior relationship in the present study (β = 0.42, *p* < 0.001; see Figure 2), it might be that the students’ habit for being physically active (i.e., past behavior) accounted for a majority of the variability in future physical activity behavior and attenuated the role of intention on behavior.

There were two posteriori effects included in the model based on suggestions by the modification indices. The first additional direct effect was from relatedness need frustration in PE to autonomous motivation toward leisure-time physical activity. According to self-determination theory [26,27], the need for relatedness is expected to be essential for continued active engagement in activities, as well as for social integration. Research has supported the vital role of students’ sense of belonging in a PE context on secure and stable interpersonal relationships outside of school [7,53]. Furthermore, good relationships with classmates in PE may particularly be exposed during the participation in physical activity outside of school with those same peers, without necessarily being related to the motivation toward PE [9]. Therefore, the reverse direct effect of perceived frustration of the need for relatedness in PE on autonomous motivation toward leisure-time physical activity found in this study is not totally surprising; that is, if students felt cold and distant relationships with their classmates in PE, the less they were motivated toward engagement in activities outside of school with those same peers.

The second posteriori effect that was included in the model based on suggestions by the modification indices was the direct effect from autonomous motivation toward leisure-time physical activity to intention with respect to the future participation in physical activity. This seems to indicate that students’ intention to participate in leisure-time physical activity in the future may also be instigated by autonomous motivation toward physical activity regardless of the formation of beliefs about physical activity. Although contrary to the hypothesis of the trans-contextual model and the current study, the direct effect of autonomous motivation toward physical activity on intention is consistent with the results of the study with the sample of middle school students [54] as well as with the sample of university students [55]. The significant direct effect of autonomous motivation toward leisure-time physical activity may reflect the more automatic, spontaneous formation of the intention for future engagement in physical activity among school students in their leisure time [55].

It is important to note that there were significant and negative indirect effects of perceived controlling behavior from PE teachers on students’ intention toward leisure-time physical activity and actual participation in physical activity. It is also worth noting that these indirect effects found in the current study, although in the opposite direction, were comparable in magnitude with the indirect effects of perceived autonomy support on students’ intention toward and actual participation in physical activity found in previous tests of the trans-contextual model [10]. Our findings indicate that students’ perceptions of controlling behavior displayed by teachers in PE play a crucial indirect role in predicting lower levels of autonomous motivation and intention toward physical activity outside of school. Another noteworthy finding was the direct decreasing effect of perceived frustration over the need for competence in PE on students’ self-reported leisure-time physical activity. This is consistent with a previous study by Koka et al. [25], who revealed a direct effect of perceived frustration over the need for competence in PE on students’ objectively measured leisure-time physical activity. These results highlight the importance of avoiding the experience of competence need frustration in PE classes, if the aim is to foster the participation in physical activity outside of school among students. From a practical perspective, PE teachers are, therefore, advised to avoid controlling behaviors while interacting with their students in classes. Behaviors that have shown to induce a controlling learning environment in PE and should be avoided include using the threat of punishment to keep students in line during lesson (i.e., intimidation), being less supportive if students are wrong or do not exercise and perform well (i.e., negative conditional regard), and using praise only to make their students exercise harder (i.e., controlling use of praise) [16,18,21,23,25].

The present study provided evidence that students’ perceptions of their teachers’ controlling behavior do not only have detrimental effects on motivation toward physical activity in PE classes, but they also have harmful effects on motivation and intention toward, and actual participation in physical activity outside of school. The strengths of this study include the application of an appropriate multi-theory-based model and the use of a prospective multi-wave design. The study, however, is not without limitations. First, although a prospective multi-wave design was adopted, the nature of the data is still correlational, which does not allow to elucidate the causal relationships between the constructs in the model. Secondly, although the attrition rate across the three waves of data collection was 18%, which is comparable with the attrition rates followed in similar studies [2,3], it still may be a cause of bias in our analyses. Moreover, attrition analyses indicated that students who dropped out had significantly higher perceptions of negative conditional regard and intimidation from PE teachers at the baseline (i.e., Time 1) than students who were retained in the final analyses. Students who dropped out from the study were also significantly younger. The reader must be, therefore, warned about the generalizability of the results to the whole student population. Thirdly, the age range of the current sample was relatively wide. One may, therefore, argue that potential behavioral- and personality-related differences between the students of different ages may lead to differences in the proposed model effects. In fact, older participants (i.e., 10th–12th graders) did have a higher intention to be physically active in their leisure time compared with younger students (i.e., 7th–9th graders). However, subsamples of the 7th–9th graders and 10th–12th graders, respectively, were not sufficiently large to meet the required minimum number of cases to parameter estimates in order to examine the invariance of the proposed model effects across these age groups. Future studies would do well by testing the invariance of the proposed model effects across different age groups. Fourthly, all variables in the hypothesized model were treated as manifest rather than latent variables. The former approach was adopted because of the complexity of the hypothesized model and relatively small sample size. Future studies with larger samples would do well by replicating the findings found in the present study. The larger sample would allow to adopt the latent variable approach, which would enable taking into account the measurement errors. Finally, it should be stressed that the results of this study are based entirely on data obtained from the participants’ self-reports. The reader must be, therefore, cautioned when interpreting the results of the present study. Despite all the measures having been used extensively in past similar studies with children and adolescents, and have demonstrated satisfactory validity and reliability, reliance entirely on self-reports should be considered as a limitation of the present study. 

## 5. Conclusions

To conclude, the findings of the present study provided evidence that students who perceive their teachers to exhibit controlling behaviors in PE classes are less likely to be autonomously motivated toward the physical activity they perform in PE classes because of a perceived frustration over the need for autonomy. More importantly, as a consequence, they are also less likely to be autonomously motivated toward the physical activity they perform outside of school, are more likely to hold maladaptive beliefs and lower intentions toward physical activity outside of school, and are more likely to report having participated less in physical activity outside of school. These findings emphasize that PE teacher training should focus on decreasing the controlling behaviors teachers may display in classes to ensure the positive impact of PE on the lifelong physical activity habits in young people. 

## Figures and Tables

**Table 1:** Descriptive statistics and reliability coefficients for each measure.

Subscale	*M*	*SD*	Skewness	Kurtosis	α
Intimidation	1.80	1.02	2.01	5.69	0.61
Controlling use of praise	3.29	1.36	0.33	−0.16	0.68
Negative conditional regard	2.64	1.32	0.86	0.61	0.65
Autonomy frustration	3.25	1.17	0.38	−0.49	0.66
Competence frustration	3.01	1.34	0.50	−0.31	0.79
Relatedness frustration	2.16	1.23	0.99	0.04	0.85
Intrinsic motivation (PE)	5.17	1.32	−0.58	0.02	0.87
Identified regulation (PE)	5.10	1.27	−0.57	−0.21	0.83
Introjected regulation (PE)	3.91	1.29	0.00	−0.51	0.64
External regulation (PE)	4.22	1.44	−0.07	−0.62	0.75
Amotivation (PE)	2.27	1.40	1.38	1.58	0.81
Intrinsic motivation (LT)	5.20	1.43	−0.72	0.05	0.90
Identified regulation (LT)	4.45	1.30	−0.42	−0.43	0.77
Introjected regulation (LT)	2.97	1.40	0.62	−0.25	0.81
External regulation (LT)	2.13	1.04	1.06	0.67	0.74
Amotivation (LT)	1.77	0.99	1.77	3.28	0.83
Attitude	5.69	1.34	−1.12	0.60	0.85
Subjective norms	3.29	1.53	0.37	−0.47	0.71
Perceived behavioral control	5.11	1.38	−0.68	−0.04	0.75
Intention	5.51	1.44	−0.71	−0.52	0.90
MVPA (MET-min/week)	2560.17	2031.52	1.23	1.26	––
Past PA	3.73	1.10	−0.15	−0.23	–

Notes: PE = physical education context; LT = leisure-time context; MVPA = moderate-to-vigorous physical activity; MET = metabolic equivalent of task.

**Table 2:** Bivariate correlations among all the study measures.

Subscale	1	2	3	4	5	6	7	8	9	10	11	12	13	14	15	16	17	18	19	20	21
1. Intimidation	–																				
2. Controlling use of praise	0.42	–																			
3. Negative conditional regard	0.64	0.52	–																		
4. Autonomy frustration	0.35	0.33	0.37	–																	
5. Competence frustration	0.26	0.26	0.34	0.65	–																
6. Relatedness frustration	0.41	0.35	0.38	0.52	0.57	–															
7. Intrinsic motivation (PE)	−0.13	−0.06	−0.09	−0.38	−0.35	−0.25	–														
8. Identified regulation (PE)	−0.09	0.04	−0.01	−0.28	−0.26	−0.12	0.80	–													
9. Introjected regulation (PE)	0.13	0.13	0.12	0.07	−0.01	0.04	0.42	0.54	–												
10. External regulation (PE)	0.33	0.33	0.38	0.43	0.38	0.24	−0.15	−0.04	0.40	–											
11. Amotivation (PE)	0.41	0.25	0.31	0.38	0.31	0.33	−0.37	−0.40	0.02	0.36	–										
12. Intrinsic motivation (LT)	−0.18	−0.07	−0.15	−0.25	−0.37	−0.32	0.52	0.46	0.23	−0.12	−0.30	–									
13. Identified regulation (LT)	−0.00	−0.00	−0.03	−0.09	−0.24	−0.19	0.48	0.51	0.40	0.01	−0.23	0.68	–								
14. Introjected regulation (LT)	0.11	0.14	0.10	0.13	0.11	0.20	0.25	0.31	0.41	0.15	−0.06	0.21	0.59	–							
15. External regulation (LT)	0.19	0.24	0.20	0.12	0.18	0.33	0.05	0.14	0.28	0.21	0.12	−0.14	0.03	0.37	–						
16. Amotivation (LT)	0.34	0.23	0.24	0.27	0.34	0.38	−0.24	−0.19	−0.00	0.16	0.37	−0.52	−0.27	0.09	0.36	–					
17. Attitude	−0.11	−0.08	−0.12	−0.23	−0.24	−0.32	0.29	0.24	0.12	−0.11	−0.20	0.54	0.45	0.14	−0.09	−0.29	–				
18. Subjective norms	0.11	0.15	0.13	0.03	0.12	0.18	0.14	0.15	0.15	0.12	0.07	−0.05	0.12	0.27	0.53	0.07	0.09	–			
19. PBC	−0.13	0.01	−0.10	−0.14	−0.24	−0.26	0.23	0.23	0.15	−0.07	−0.16	0.46	0.39	0.05	−0.13	−0.30	0.52	0.12	–		
20. Intention	−0.07	−0.04	−0.09	−0.15	−0.24	−0.32	0.30	0.27	0.18	−0.03	−0.18	0.56	0.51	0.13	−0.14	−0.41	0.61	0.16	0.69	–	
21. MVPA (MET-min/week)	−0.07	−0.00	−0.09	−0.13	−0.24	−0.11	0.14	0.14	0.12	−0.05	−0.07	0.29	0.24	0.09	−0.09	−0.23	0.16	0.11	0.24	0.29	–
22. Past PA	−0.03	0.10	0.05	−0.10	−0.17	−0.08	0.28	0.37	0.24	−0.04	−0.16	0.45	0.48	0.26	−0.07	−0.29	0.35	0.10	0.45	0.48	0.46

Notes: PE = physical education context; LT = leisure-time context; PBC = perceived behavioral control; MVPA = moderate-to-vigorous physical activity; MET = metabolic equivalent of task. Bivariate correlations of 0.13 and above are significant at the *p* < 0.05 level; bivariate correlations of 0.17 and above are significant at the *p* < 0.01 level.

**Table 3:** Standardized indirect effects in the model.

Parameter	β CI 95% (Lower, Upper)	Parameter	β CI 95% (Lower, Upper)
Controlling behavior from teachers → SDI-PE	−0.24 *** (−0.34, −0.15)	Competence need frustration → MVPA	−0.00 (−0.01, 0.00)
Controlling behavior from teachers → SDI-LT	−0.21 *** (−0.31, −0.13)	Relatedness need frustration → SDI-LT	−0.01 (−0.06, 0.02)
Controlling behavior from teachers → Attitude	−0.09 *** (−0.16, −0.05)	Relatedness need frustration → Attitude	−0.15 *** (−0.23, −0.08)
Controlling behavior from teachers → Subjective norm	0.06 *** (0.03, 0.10)	Relatedness need frustration → Subjective norm	0.10 *** (0.05, 0.16)
Controlling behavior from teachers → PBC	−0.07 *** (−0.12, −0.04)	Relatedness need frustration → PBC	−0.12 *** (−0.19, −0.06)
Controlling behavior from teachers → Intention	−0.10 *** (−0.17, −0.06)	Relatedness need frustration → Intention	−0.16 *** (−0.25, −0.10)
Controlling behavior from teachers → MVPA	−0.05 ** (−0.11, −0.01)	Relatedness need frustration → MVPA	−0.01 (−0.03, 0.01)
Autonomy need frustration → SDI-LT	−0.10 *** (−0.16, −0.06)	SDI-PE → Attitude	0.11 *** (0.06, 0.18)
Autonomy need frustration → Attitude	−0.04 *** (−0.08, −0.02)	SDI-PE → Subjective norm	−0.07 *** (−0.13, −0.04)
Autonomy need frustration → Subjective norm	0.03 *** (0.01, 0.05)	SDI-PE → PBC	0.09 *** (0.05, 0.15)
Autonomy need frustration → PBC	−0.04 *** (−0.06, −0.02)	SDI-PE → Intention	0.12 *** (0.07, 0.19)
Autonomy need frustration → Intention	−0.05 *** (−0.08, −0.03)	SDI-PE → MVPA	0.01 (−0.01, 0.03)
Autonomy need frustration → MVPA	−0.00 (−0.01, 0.00)	SDI-LT → Intention	0.20 *** (0.10, 0.31)
Competence need frustration → SDI-LT	−0.04 (−0.09, 0.00)	SDI-LT → MVPA	0.03 (−0.03, 0.09)
Competence need frustration → Attitude	−0.02 (−0.04, 0.00)	Attitude → MVPA	0.02 (−0.01, 0.06)
Competence need frustration → Subjective norm	0.01 ^a^ (0.00, 0.03)	Subjective norm → MVPA	0.01 (−0.00, 0.04)
Competence need frustration → PBC	−0.01 (−0.04, 0.00)	PBC → MVPA	0.03 (−0.02, 0.09)
Competence need frustration → Intention	−0.02 (−0.05, 0.00)		

Notes: CI = confidence intervals; SDI-PE = self-determination index in a physical education context; SDI-LT = self-determination index in a leisure-time context; PBC = perceived behavioral control; MVPA = moderate-to-vigorous physical activity. ** *p* < 0.01; *** *p* < 0.001; ^a^ *p* = 0.05.

**Table 4:** Specific indirect effects from perceived controlling behavior from teachers on students’ intention to be physically active in their leisure time.

Specific Indirect Effects	B 95% CI (Lower, Upper)	β
Controlling behavior from teachers → Autonomy need frustration → SDI-PE → SDI-LT → Intention	−0.024 *** (−0.052, −0.009)	−0.012
Controlling behavior from teachers → Autonomy need frustration → SDI-PE → SDI-LT → Attitude → Intention	−0.009 *** (−0.022, −0.003)	−0.004
Controlling behavior from teachers → Autonomy need frustration → SDI-PE → SDI LT → Subjective norm → Intention	0.003 ** (0.001, 0.008)	0.001
Controlling behavior from teachers → Autonomy need frustration → SDI-PE → SDI-LT → Perceived behavioral control → Intention	−0.011 *** (−0.025, −0.005)	−0.006
Controlling behavior from teachers → Relatedness need frustration → SDI-LT → Intention	−0.084 *** (−0.160, −0.035)	−0.043
Controlling behavior from teachers → Relatedness need frustration → SDI-LT → Attitude → Intention	−0.031 *** (−0.069, −0.011)	−0.015
Controlling behavior from teachers → Relatedness need frustration → SDI-LT → Subjective norm → Intention	0.010 ** (0.003, 0.026)	0.005
Controlling behavior from teachers → Relatedness need frustration → SDI-LT → Perceived behavioral control → Intention	−0.039 *** (−0.079, −0.018)	−0.020

Notes: CI = confidence intervals; SDI-PE = self-determination index in a physical education context; SDI-LT = self-determination index in a leisure-time context. ** *p* < 0.01; *** *p* < 0.001.
